# Neighborhood Environment and Falls among Community-Dwelling Older Adults

**DOI:** 10.3390/ijerph14020175

**Published:** 2017-02-10

**Authors:** Emily Joy Nicklett, Matthew C. Lohman, Matthew Lee Smith

**Affiliations:** 1 School of Social Work, The University of Michigan, Ann Arbor, MI 48109, USA; 2 Department of Psychiatry, Dartmouth Geisel School of Medicine, Lebanon, NH 03766, USA; matt.lohman@gmail.com; 3 Department of Health Promotion and Behavior, Institute of Gerontology, College of Public Health, University of Georgia, Athens, GA 30602, USA; health@uga.edu; 4 Department of Health Promotion and Community Health Sciences, School of Public Health, Texas A&M University, College Station, TX 77842, USA

**Keywords:** neighborhood factors, social cohesion, physical disorder, older adults, fall events

## Abstract

*Background*: Falls present a major challenge to active aging, but the relationship between neighborhood factors and falls is poorly understood. This study examined the relationship between fall events and neighborhood factors, including neighborhood social cohesion (sense of belonging, trust, friendliness, and helpfulness) and physical environment (vandalism/graffiti, rubbish, vacant/deserted houses, and perceived safety walking home at night). *Methods*: Data were analyzed from 9259 participants over four biennial waves (2006–2012) of the Health and Retirement Study (HRS), a nationally representative sample of adults aged 65 and older in the United States. *Results*: In models adjusting for demographic and health-related covariates, a one-unit increase in neighborhood social cohesion was associated with 4% lower odds of experiencing a single fall (odds ratio (OR): 0.96, 95% confidence interval (CI): 0.93–0.99) and 6% lower odds of experiencing multiple falls (OR: 0.94, 95% CI: 0.90–0.98). A one-unit increase in the physical environment scale was associated with 4% lower odds of experiencing a single fall (OR: 0.96, 95% CI: 0.93–0.99) and with 5% lower odds of experiencing multiple falls (OR: 0.95, 95% CI: 0.91–1.00) in adjusted models. *Conclusions*: The physical and social neighborhood environment may affect fall risk among community-dwelling older adults. Findings support the ongoing need for evidence-based fall prevention programming in community and clinical settings.

## 1. Introduction

Falls are the leading cause of injury-related mortality among adults aged 65 and older. According to national surveillance estimates, falls are responsible for approximately seven million injuries per year among older adults in the United States [1,2]. Fall-related injuries contribute to physical limitations, disability, and restricted activities of daily living [3]. The consequences of falls and their aftermath can be self-perpetuating due to activity avoidance and subsequent deconditioning [4,5]. Further, fear of falling might prevent older adults from engaging in health-promoting behaviors and activities [6]. The majority of older adults want to remain in their own home or community for as long as possible [7]. Falls are a major concern for older adults who aspire to age in place.

### 1.1. The World Health Organization: Four Pillars of Fall Prevention 

The World Health Organization (WHO) identifies four categories of risk factors for falls in older age: (1) biological risk factors (including age, gender, race, chronic illnesses, and physical or cognitive decline); (2) behavioral risk factors (such as multiple medication use, excess alcohol intake, and lack of exercise); (3) social and economic risk factors (including socioeconomic resources, access to adequate housing and community resources/services, and social interaction); and (4) environmental risk factors, which can be within homes or in the neighborhood environment (such as poor building design, slippery floors and stairs, looser rugs, insufficient lighting, and cracked or uneven sidewalks) [8].

An extensive literature characterizes the relationship between falls and biological, behavioral, and—to some extent—social and economic risk factors. This research informs clinical practice and community-based fall prevention programs through targeted intervention strategies [9,10,11,12,13,14]. The accuracy of fall-risk assessment tools varies by setting and context [15]. Fall prevention programs therefore tend to be more effective when they are multi-factorial [16], community-based [17,18], and specifically address hazards and risk factors in the environment [11,19,20]. However, very few fall prevention programs or interventions involve modifying aspects of the community environment to reduce the risk of falls in the United States [10]. One intervention program targeted perceived environmental hazards (e.g., improved lighting, sidewalk maintenance, winter road maintenance), which was associated with a 26% reduction in falls among adults aged 65–79 in Sweden [19]. Further evidence is needed about neighborhood-level risk and protective factors for falls among older adults in the United States to inform clinical practice and interventions. 

### 1.2. Neighborhood Factors and Falls among Older Adults

Older adults spend relatively more time in their residential neighborhoods for social, recreational, and task-related activities compared to younger age groups [21]. Approximately half of falls occur outside the home [22]. Despite the strong potential impact of improved identification of modifiable, community-level determinants of falls, relatively few studies have examined how neighborhood factors relate to falls among older adults specifically [19,22,23,24,25,26,27,28]. Socially cohesive neighborhoods, those in which older adults reported high levels of belonging and trust, entail a sense of place in one’s community, as opposed to feelings of isolation or social dislocation. Neighborhood physical environments also affect the well-being of older adults through accessibility, safe paths, and aesthetically-pleasing destinations.

Several recent studies have examined the role of neighborhood factors in fall events, particularly if they operate as risk and protective factors for fall events. A study by Li and colleagues [22] examined past-year falls among 2193 middle-aged and older adults in Northern California between 1996 and 2001. They found that most outdoor falls occurred during walking (47.3%) and that the majority of outdoor falls took place on sidewalks, curbs, and streets (73%) [22]. A qualitative study authored by Chippendale and Boltz [23] identified perceived fall risk among 14 community-dwelling older adults. They found that older adults were less likely to walk in areas they considered unsafe [23] but that they were more likely to walk if they perceived others would be available to lend a hand at pedestrian crossings or in hazardous walking conditions [23]. This emerging research suggests that the neighborhood or community context in which falls occur is integral to understanding fall events among community-dwelling older adult populations [8,11,22,24,26,27].

Further research is needed to examine neighborhood-level predictors of fall events among community-dwelling older adults in the United States. The existing body of literature on environmental determinants of falls has limited generalizability to U.S. older adults. Challenges to external validity of existing studies include small sample sizes and non-representative convenience samples [23,24], and cross-sectional data or short follow-up periods [25,27,29]. Most studies examining neighborhood factors and falls were conducted outside of the United States [19,24,25,27] or in defined geographic regions not generalizable to the U.S. population [22,23].

The present study describes the relationship between perceptions of the neighborhood environment and falls among community-dwelling older adults, using a nationally representative sample of community-dwelling older adults. We tested two hypotheses. First, we hypothesized that perceived neighborhood cohesion is associated with lower risk of experiencing fall events, after adjusting for sociodemographic and health-related characteristics. Second, we hypothesized that neighborhood physical environments—such as the presence or absence of hazards—are associated with fall events, after adjusting for sociodemographic and health-related characteristics. We also examined which aspects of the neighborhood environment specifically related to higher or lower fall risk.

## 2. Materials and Methods 

### 2.1. Sample

Data were examined from the Health and Retirement Study (HRS), a national longitudinal survey of older Americans that began in 1992. The primary HRS questionnaire, administered every two years, asks participants to report detailed information regarding demographic, social, economic, and health characteristics. The 2006 HRS wave included interviews from 18,469 respondents. Beginning in 2006, half of HRS respondents were randomly selected to participate in enhanced face-to-face interviews, including an additional leave-behind questionnaire, which asked respondents to report levels of social participation, neighborhood characteristics, psychological well-being, and other psychosocial variables. Respondents were eligible for enhanced interviews if they were alive and non-institutionalized at the time of the interview.

Data were examined from respondents aged 65 and older interviewed in the 2006, 2008, 2010, and/or 2012 HRS waves (*n* = 12,311). We excluded participants who did not provide answers to questions related to falls at any wave (*n* = 15) and participants who were not selected for at least one leave-behind psychosocial questionnaire (*n* = 64). Of the remaining 12,232 respondents, 790 were ineligible for the leave-behind psychosocial questionnaire at the time of interview and 265 responded by proxy. Of the remaining eligible participants, the leave-behind questionnaire was not administered to 924 participants. Further, 994 completed the leave-behind questionnaire, but had missing data for the psychosocial measures. After applying these inclusion/exclusion criteria, the analytical sample was restricted to 9259 non-proxy respondents aged 65 and older who completed at least one psychosocial questionnaire during the study period. 

### 2.2. Measures

*Outcomes*. Fall events (any falls, multiple falls) were reported by respondents at each biennial wave in the core HRS questionnaire (2006–2012). Respondents were asked whether they had fallen in the previous two-year period and, if so, how many times they had fallen during that period. We considered two outcome events in separate analyses—*any fall*, defined as reporting at least one fall during the past two years, and *multiple falls*, defined as two or more falls during the past two years. This strategy was informed by prior research on risk and protective factors for fall events [13,16]. Both outcome variables were operationalized as binary variables (reported outcome vs. did not report the outcome) in analysis. These outcomes include indoor and outdoor falls [28].

*Neighborhood environment*. Neighborhood environment is examined using neighborhood social cohesion and neighborhood physical environment scales. Participants were asked about features of their local community or “everywhere within a 20-min walk or about a mile of your home”. The *neighborhood social cohesion* scale [30] examines the extent to which participants: (1) consider themselves part of their area; (2) trust most people in their area; (3) consider most people in their area friendly; and (4) believe that people in their area would help if they were in trouble. The *neighborhood physical environment* scale (adapted from a neighborhood physical disorder scale [30,31,32,33,34]) examines the extent to which participants: (1) consider vandalism and graffiti to be a problem in their area; (2) feel safe walking alone after dark; (3) consider their area kept clean and relatively absent of litter/rubbish; and (4) consider their area absent of vacant or deserted houses or storefronts. 

Both scales were constructed using an index of average scores for four items, each with a seven-point scale (range 1–7). Higher values on the scale indicate greater perceived neighborhood social cohesion and the absence of perceived neighborhood physical disorder or hazards. For each scale, responses to the four items were averaged to create individual-level measures of perceived neighborhood social cohesion and neighborhood physical environment. Final scores were set to missing if two or more items in the scale were missing (*n* = 184) [30]. The neighborhood social cohesion and physical environment scales had associated Cronbach’s alpha scores of 0.86 and 0.82, respectively, based on the 2010 wave of the study [30].

*Control variables*. We included fall-related risk factors as covariates that could confound the examined relationship between environmental risk factors and fall events. Following prior research and the WHO framework of fall prevention in older age [8], our control variables are in the remaining three domains: biological risk factors, behavioral risk factors, and social/economic risk factors.

Biological factors included older age [9], female sex [35,36], co-occurring chronic health conditions [35,37], dizziness/syncope [38], and functional limitations/decline [39]. Behavioral risk factors included physical activity behavior, which is associated with lower risk of falls [10,40] and is a leading cause of fall events among older adults [22]. We also considered social and economic characteristics that could confound the relationship between neighborhood social conditions and falls, including individual-level socioeconomic status/financial resources [9,41], marital status, and racial/ethnic group [13]. 

The relationships between fall events and included control variables are shown in Table 1. For multivariate analyses, we classified covariates as demographic characteristics or health-related characteristics. *Demographic characteristics* included socioeconomic status (i.e., years of education and wealth), age and age group (i.e., 65–69; 70–74; 75–79; 80–84; 85 or older), sex (i.e., male, female), marital status (i.e., whether married or partnered, divorced/separated/never married, or widowed), and race/ethnicity (i.e., non-Hispanic white, non-Hispanic black, Hispanic/Latino, other). Wealth was measured as total assets (including home wealth) minus total debts [42] and was log-transformed.

*Health-related characteristics* included co-occurring chronic conditions, physical activity level, whether the respondent had ever experienced dizziness, and Activities of Daily Living (ADL) limitations. We created a binary variable for some difficulty with ADLs, indicating whether participants reported having “at least some difficulty” in independently doing one or more of the following activities: walking across a room, getting in and out of bed, dressing, bathing, and eating. The number of co-occurring chronic conditions was determined by the number of conditions with which respondents reported having been diagnosed—including high blood pressure, diabetes, cancer, lung disease, heart problems, stroke, and arthritis. The physical activities score is a weighted sum of self-reported frequency of vigorous, moderate, and light activity levels. Activity level was weighted by average Metabolic Equivalency of Task scores: light activity (1–2.9 METS for an average of 2), moderate activity (3–5.9 METs for an average of 4.5) and vigorous activity (6–10 METS for an average of 8) [43]. 

### 2.3. Data Analysis

We generated summary descriptive statistics comparing demographic, psychosocial, and health-related characteristics, overall and by fall outcome status. To estimate the association between neighborhood characteristics and falls, we first fit crude longitudinal logistic mixed-effects models of fall likelihood, including participant-level random intercepts with unstructured covariance and fixed effects for time (HRS wave) and neighborhood social cohesion. Potential for confounding was assessed using a change-in-estimate procedure in which potential confounding variables were added individually to crude models for adjustment. Covariates producing a 10% or greater change in the estimated association between falls and neighborhood social cohesion comparing crude and adjusted models were considered significant confounders. Because no individual covariates were found to be significant confounders using the 10% change-in-estimate criterion, we fit three alternative adjusted models, adjusting respectively for sociodemographic characteristics only, sociodemographic and health-related characteristics, and for sociodemographic and health-related characteristics including cognitive and depressive symptom level. Model fitting steps were repeated with neighborhood physical environment as the primary independent outcome.

All regression models and statistical analyses were performed using STATA (v.14) statistical software (StataCorp, College Station, TX, USA). Of the 9259 respondents in the analytic sample, 70% (6505) had falls data for all 4/4 waves, 13.2% (1221) had falls data for 3/4 waves, 12.1% (1123) had falls data for 2/4 waves, and only 4.4% (410) had falls data for only 1/4 waves. Thus, in total, only 7.4% (2754/37,036) of possible fall measures were missing due to attrition over time or other reasons. On average, respondents had measures for the fall outcome at 3.5/4 waves. Missing data for all covariates included in adjusted models was <5% and so was also unlikely to have significantly influenced results. All models were estimated using maximum likelihood methods (likelihood approximated using adaptive Gauss-Hermite quadrature) and robust variance estimation.

## 3. Results

Descriptive statistics of neighborhood characteristics and demographic/health-related covariates and fall events are shown in Table 1. 

The average age of participants was 73.86 (±6.88) years. The majority of participants (57.2%) were female and most identified as non-Hispanic white (78.5%), followed by African American (12.3%), Hispanic/Latino (6%), and other racial/ethnic group (3.1%). Among participants, total wealth (excluding all debts) ranged from −$865,000 to $42,500 (quintile 1) to $718,000 to over $1 million (quintile 5). Chronic illness comorbidity was highly prevalent among fallers and participants who experienced multiple falls were more likely to have each of the seven examined chronic illnesses (i.e., high blood pressure, diabetes, cancer, lung disease, heart problems, stroke, and arthritis) than those who experienced single fall events or no falls (analyses not shown; descriptive statistics of baseline study characteristics and fall events are reported in Table 1).

At baseline, 35% of respondents reported no falls in the past two years, while 24% reported a single fall and 41% reported multiple falls. Participants who had not fallen or who had experienced a single fall reported, on average, higher levels of perceived neighborhood cohesion, while participants who had fallen multiple times reported relatively lower levels of neighborhood cohesion (*p* < 0.05). Participants who had experienced multiple falls reported a relatively greater presence of potential hazards in the neighborhood physical environment, such as presence of rubbish and feeling unsafe walking alone at night (*p* < 0.05). 

### Multivariate Analyses

**Neighborhood social cohesion.** Table 2 summarizes results from analyses of neighborhood social cohesion and fall events, which addresses the first hypothesis. Participants who perceived their neighborhoods as more cohesive experienced fewer falls. Each unit increase in average neighborhood social cohesion was associated with 6% lower odds of experiencing a fall (OR: 0.94, 95% CI: 0.91–0.97) and 9% lower odds of experiencing multiple falls (OR: 0.91, 95% CI: 0.87–0.95) in unadjusted models. In models adjusting for sociodemographic characteristics, each unit increase was associated with 7% lower odds of experiencing a fall (OR: 0.93, 95% CI: 0.90–0.96) and 10% lower odds of experiencing multiple falls (OR: 0.90, 95% CI: 0.86–0.94). In fully-adjusted models, which control for health-related covariates, each unit increase in the average social cohesion scale was associated with 4% lower odds of experiencing a fall (OR: 0.96, 95% CI: 0.93–0.99) and 6% lower odds of experiencing multiple falls (OR: 0.94, 95% CI: 0.90–0.98). For example, participants who reported an average score of 7 versus 4 on the neighborhood social cohesion scale were 20% less likely to experience any falls and were 27% less likely to experience multiple falls (in models adjusting for sociodemographic characteristics). These results offer support to the hypothesized relationship between social cohesion and falls.

**Neighborhood physical environment.** Table 3 summarizes results from analyses of the neighborhood physical environment and fall events, which concerns the second hypothesis. Participants who perceived their neighborhood physical environments as favorable experienced fewer falls. Each unit increase in average neighborhood environment score was associated with 5% lower odds of experiencing a fall (OR: 0.95, 95% CI: 0.92–0.98) and 7% lower odds of experiencing multiple falls (OR: 0.93, 95% CI: 0.89–0.97) in unadjusted models. In models adjusting for sociodemographic characteristics, each unit increase was associated with 6% lower odds of experiencing a fall (OR: 0.94, 95% CI: 0.91–0.97) and 8% lower odds of experiencing multiple falls (OR: 0.92, 95% CI: 0.88–0.96). In fully-adjusted models, which control for health-related covariates, each unit increase in the average physical environment scale was associated with 4% lower odds of experiencing a fall (OR: 0.96, 95% CI: 0.93–0.99) and 5% lower odds of experiencing multiple falls (OR: 0.95, 95% CI: 0.91–1.00). For example, participants who reported an average score of 7 versus 4 on the neighborhood physical environment scale were 17% less likely to experience any falls and were 22% less likely to experience multiple falls (in models adjusting for sociodemographic characteristics). These results offer support to the hypothesized relationship between absence of hazards and other features of the physical environment and falls. 

**Specific neighborhood factors.** The relationships between specific neighborhood factors and fall events are shown in Table 4. 

Among the examined neighborhood social cohesion measures, higher levels of agreement with the statements “I feel part of this area” (OR: 0.95, 95% CI: 0.93–0.98) and “People in this area will help you” (OR: 0.97, 95% CI: 0.94–1.00) were associated with lower odds of experiencing any falls, adjusting for sociodemographic and health-related covariates. Higher levels of agreement with the statements “I feel part of this area” (OR: 0.95, 95% CI: 0.92–0.99) and “I trust people in this area” (0.96, 95% CI: 0.92–0.99) were associated with lower odds of experiencing multiple falls in fully-adjusted models. Among neighborhood social cohesion measures, perceived friendliness was not significantly associated with fall events (any or multiple) in fully-adjusted models (at *p* < 0.05). 

Examined factors related to the neighborhood physical environment also varied in their association with single and/or multiple fall events. Perceived absence of rubbish (OR: 0.97, 95% CI: 0.94–1.00) and perceived safety walking alone at night (OR: 0.97, 95% CI: 0.95–1.00) were associated with lower odds of experiencing any falls, adjusting for sociodemographic and health-related covariates. Participants reporting less rubbish in their neighborhoods were also less likely to experience multiple falls (OR: 0.95; 95% CI: 0.91–0.99). Among neighborhood physical environment measures, presence/absence of vacant/deserted homes and vandalism/graffiti were not significantly associated with fall events in the fully-adjusted models (at *p* < 0.05).

## 4. Discussion

The aim of this study was to identify the relationships between perceived neighborhood environment and falls among a nationally representative sample of community-dwelling older adults aged 65 and older. The results of this study supported the hypotheses that the social and physical characteristics of one’s neighborhood environment relate to falls among community-dwelling older adults in the United States. First, this study found that higher perceived neighborhood cohesion was associated with lower likelihood of falls. Second, this study found that features of the neighborhood physical environment were associated with fall events. Finally, this study identified relationships between specific neighborhood characteristics and fall events. Factors in the neighborhood environment associated with fall events included perceived neighborhood belonging, trust, and willingness of others to help. Factors in the social environment associated with fall events included the relative perceived absence of rubbish and feeling safe walking alone at night. 

These findings make contributions to the literature about neighborhood determinants of health among older adult populations. In prior studies, neighborhood social cohesion has been linked to improved self-rated health/subjective well-being [44,45] and lower frailty risk [46] among older adults. Neighborhood physical environment features have also been linked to walking behaviors and other forms of physical activity among older adults [23,31].

The underlying relationships between falls and neighborhood characteristics likely involve direct and indirect processes. Neighborhood environments may influence fall risk directly, for example, due to trips or slips on uneven surfaces or objects [22]. The relationship could also operate through relative access to health care services, commerce, physical activity, or meaningful social interaction. In this study, two specific neighborhood physical features—perceived safety walking at night and the presence of rubbish in the neighborhood—were independently related to fall risk. These findings are consistent with prior studies that found the presence or absence of neighborhood safety and environmental hazards to affect engagement in neighborhood physical [23] and social activity [47] among older adults. Of note, we found that these factors were related to fall risk even after accounting for different levels of physical activity. Another potential explanation is that neighborhood safety concerns and poor appearance suggest less civic investment and fewer socioeconomic resources that could improve neighborhood safety and livability [48,49]. For instance, previous research suggests that a lack of neighborhood resources for safe walking spaces, sidewalk repair, benches, unreliable public transit, and other features may contribute directly to both the opportunity for and likelihood of falls [21]. Future studies should examine the specific relationship between neighborhood socioeconomic levels and fall risks. 

In this study, several aspects of the neighborhood social environment were independently associated with fall risk: sense of belonging in one’s neighborhood, trusting neighbors, and willingness of others to help when needed. Consistent with prior research by Kawachi and colleagues [48,50], several aspects of neighborhood social cohesion were associated with fewer adverse health effects, in this case falls. Perceived cohesion, belonging, and trust are a complex function of objective neighborhood qualities, subjective assessment of neighborhood characteristics, personal feelings of safety, and quality of individual social contacts [46]. Individuals’ sense of belonging in their neighborhoods might therefore represent the influence of physical neighborhood features in fostering healthy social contacts, as well as the support and resources provided by robust social networks. Older adults who report a high sense of belonging and trust in neighbors may be more willing to rely on neighbors for support and assistance with mobility and functional tasks, especially in times of need, such as lending a hand when walking conditions are hazardous. A high sense of trust and belonging also encourages physical and social activities among older adults such as neighborhood walking activity [51], which promotes cognitive function [52] and mobility [53], two major determinants of falls [54,55]. Stress and adaptation could also play a role, with negative neighborhood physical features serving as ambient stressors and elements of social cohesiveness acting as resources to help cope with and adapt to those stressors [50]. In this conceptual model, the interplay and balance of neighborhood-level stress and coping resources may have direct influence on one’s health and well-being, which intersect with—and compounds—vulnerability to fall events in older age. 

Features of the social and physical environment could affect fall risk in myriad ways. Some neighborhood factors—such as perceived safety, walkability, and social interaction—encourage involvement in physical and social activities outside the home, which is associated with better health and well-being in later life. Consistent with prior studies [40], we found that more active older adults were less likely to fall. However, among older adults, outdoor fall events often occur during sports and activities, such as walking [21]. Environmental hazards confronted during these activities are an underlying cause for injurious outdoor falls [21]. Other neighborhood factors—such as perceived crime and environmental hazards—can lead to activity avoidance due to fear of falling and subsequent deconditioning, which could in turn increase the risk of indoor falls [4,6,56]. In sum, the relationship between neighborhood factors and falls could differ in magnitude and direction for indoor versus outdoor falls. Additional research is needed to examine risk and protective factors for indoor and outdoor falls. 

The present study builds on previous work on neighborhood and social environments and fall risk [22,23] and identifies potential targets for efforts to prevent or reduce fall risk. While many fall prevention interventions focus on enhancing personal fitness, physical activity, nutrition, or home modifications, these findings suggest that neighborhood and social context are also important elements to consider [57,58]. For instance, municipal efforts to promote safe walking spaces, installation of benches, bike paths, and other improvements may supplement programs aimed at directly stimulating physical activity among older adults, as well as remove or mitigate neighborhood hazards. Few studies have examined the influence of neighborhood safety improvements on fall risk; however, limited research suggests that such improvements may decrease the fall occurrence among some older adults [19]. Community-based intervention strategies have also been linked to greater intended activity and mobility control [56]. Likewise, fall prevention strategies that encourage social participation in group-based activities might offer more benefit than individually-targeted strategies [20], which can foster sense of community trust and belonging. Such interventions may more effectively foster social cohesion if they are offered in a variety of settings close to where the participants reside [59,60,61], thus forming or strengthening existing bonds and trust.

To our knowledge, this is the first study to examine the relationships between neighborhood characteristics and falls longitudinally among a geographically diverse, population-based sample of community-dwelling older adults in the United States. The findings of this study suggest that, consistent with the WHO framework [8], the neighborhood environment provides an important context for examining fall-related risk and protective factors. However, several limitations should be considered alongside these findings. Specifically, we were not able to examine where fall events occurred—whether indoors or outdoors—and which circumstances or activities provoked fall events. Several features of the neighborhood physical environment that have been previously linked to outdoor falls, including insufficient lighting, cracked or uneven sidewalks, and weather-related hazards [8,22,23], were not included among our measures. Further, we were not able to examine features of the home environment that have been previously linked to falls, including building design, loose rugs, and slippery floors and stairs [8,27,28]. These unexamined factors in the home and community environments might affect the magnitude and direction of examined relationships. Finally, fall events and perceived neighborhood factors are based on self-report. To inform fall prevention and intervention strategies, future research should identify modifiable risk factors for falls in the home and community environments in which they occur. 

## 5. Conclusions

Fall events could be a potential mechanism through which neighborhood environments affect the health and well-being of older adults, including opportunities to age in place. Intervention strategies that improve perceived neighborhood safety, trust, and address trip hazards could reduce the risk of outdoor falls and encourage community social engagement and physical activity, which promote health and well-being among older adults. Future research should examine the role of socioeconomic factors, physical activity, and social engagement in the relationship between neighborhoods and falls.

## Figures and Tables

**Table 1:** Baseline characteristics of the study population, *n* = 9259.

Characteristics	Total *n* = 9259 *n* (%)	Composite Outcome *			
		No Falls *n* = 3205 *n* (%)	Single Fall *n* = 2219 *n* (%)	Multiple Falls *n* = 3835 *n* (%)	*p*-Value ^†^
**Neighborhood social cohesion (total)**	5.55 (1.41)	5.59 (1.42)	5.66 (1.35)	5.46 (1.43)	<0.01
I feel a part of this area	5.54 (1.75)	5.62 (1.72)	5.59 (1.72)	5.45 (1.80)	<0.01
I trust people in this area	5.58 (1.70)	5.58 (1.71)	5.71 (1.61)	5.51 (1.74)	<0.01
People in this area are friendly	5.71 (1.67)	5.73 (1.65)	5.81 (1.59)	5.62 (1.72)	<0.01
People in this area will help you	5.38 (1.41)	5.43 (1.64)	5.50 (1.62)	5.28 (1.71)	<0.01
**Neighborhood physical environment (total)**	5.48 (1.40)	5.49 (1.43)	5.57 (1.34)	5.42 (1.40)	<0.01
Absence of vandalism/graffiti	5.55 (1.87)	5.56 (1.86)	5.60 (1.82)	5.50 (1.91)	0.10
Absence of rubbish	5.66 (1.66)	5.68 (1.64)	5.77 (1.59)	5.58 (1.70)	<0.01
Absence of vacant/deserted houses	5.66 (1.88)	5.66 (1.90)	5.71 (1.85)	5.63 (1.89)	0.20
Safe walking alone at night	5.06 (2.00)	5.07 (2.00)	5.21 (1.94)	4.97 (2.03)	<0.01
**Demographic characteristics**					
Years of education	12.25 (3.20)	12.29 (3.17)	12.43 (3.11)	12.12 (3.26)	<0.01
Age (years)	73.86 (6.88)	72.74 (6.41)	73.84 (6.82)	74.82 (7.14)	<0.01
Female (%)	5300 (57.24)	1669 (52.07)	1382 (62.28)	2249 (58.64)	<0.01
**Wealth (log-transformed)**					<0.01
Quintile 1 (lowest)	1852 (20.00)	597 (18.63)	413 (18.61)	842 (21.96)	
Quintile 2	1852 (20.00)	592 (18.47)	441 (19.87)	819 (21.36)	
Quintile 3	1859 (20.08)	677 (21.12)	446 (20.10)	736 (19.19)	
Quintile 4	1847 (19.95)	654 (20.41)	468 (21.09)	725 (18.90)	
Quintile 5 (highest)	1849 (19.97)	685 (21.37)	451 (20.32)	713 (18.59)	
**Marital status**					<0.01
Married	5781 (62.44)	2096 (65.40)	1368 (61.65)	2317 (60.42)	
Not married	1042 (11.25)	378 (11.79)	263 (11.85)	401 (10.46)	
Widowed	2436 (26.31)	731 (22.81)	588 (26.50)	1117 (29.13)	
**Race/ethnicity**					<0.01
White (non-Hispanic)	7271 (78.53)	2406 (75.07)	1759 (79.27)	3106 (80.99)	
Black (non-Hispanic)	1140 (12.31)	467 (14.57)	277 (12.48)	396 (10.33)	
Hispanic/Latino	558 (6.03)	206 (6.43)	114 (5.14)	238 (6.21)	
Other	290 (3.13)	126 (3.93)	69 (3.11)	95 (2.48)	
**Health-related covariates**					
Co-occurring conditions	2.15 (1.27)	1.91 (1.21)	1.96 (1.20)	2.45 (1.30)	<0.01
Dizziness	1120 (12.11)	252 (7.87)	225 (10.15)	643 (16.78)	<0.01
ADL difficulty	1487 (16.07)	292 (9.11)	261 (11.77)	934 (24.35)	<0.01
**Regular physical activity**					<0.01
Quintile 1 (lowest)	1880 (20.30)	506 (15.79)	386 (17.40)	988 (25.76)	
Quintile 2	2193 (23.69)	762 (23.78)	508 (22.89)	923 (24.07)	
Quintile 3	1502 (16.22)	511 (15.94)	386 (17.40)	605 (15.78)	
Quintile 4	1842 (19.89)	706 (22.03)	471 (21.23)	665 (17.34)	
Quintile 5 (highest)	1842 (19.89)	720 (22.46)	468 (21.09)	654 (17.05)	

* Fall Events; **^†^** Chi-square test (categorical), ANOVA test (means).

**Table 2:** Neighborhood social cohesion and odds of falls using mixed-effect logistic regression models (robust variance estimation), *n* = 9259.

Characteristics	Any Fall			Multiple Falls		
	cOR	aOR ^1^	aOR ^2^	cOR	aOR ^1^	aOR ^2^
**Neighborhood social cohesion**	0.94 (0.91–0.97)	0.93 (0.90–0.96)	0.96 (0.93–0.99)	0.91 (0.87–0.95)	0.90 (0.86–0.94)	0.94 (0.90–0.98)
**Time (HRS wave)**	1.07 (1.05–1.09)	1.08 (1.06–1.10)	1.10 (1.08–1.12)	1.13 (1.10–1.16)	1.14 (1.11–1.17)	1.16 (1.13–1.19)
**Demographic characteristics**						
Years of education		0.98 (0.97–1.00)	1.01 (0.99–1.03)		0.97 (0.94–0.99)	1.00 (0.97–1.03)
Wealth (log-transformed)		0.65 (0.49–0.86)	0.89 (0.69–1.16)		0.62 (0.40–0.96)	1.06 (0.71–1.57)
**Age group**						
65–69		1.00	1.00		1.00	1.00
70–74		1.20 (1.05–1.37)	1.15 (1.01–1.30)		1.28 (1.07–1.54)	1.21 (1.01–1.46)
75–79		1.64 (1.42–1.90)	1.44 (1.25–1.67)		1.77 (1.44–2.17)	1.45 (1.19–1.77)
80–84		1.76 (1.48–2.10)	1.51 (1.27–1.79)		1.81 (1.43–2.30)	1.42 (1.12–1.80)
85 and older		2.40 (1.95–2.95)	1.84 (1.49–2.27)		2.81 (2.12–3.72)	1.96 (1.46–2.62)
**Sex**						
Male		1.00	1.00		1.00	1.00
Female		1.22 (1.09–1.36)	1.21 (1.08–1.35)		1.03 (0.89–1.19)	1.01 (0.87–1.17)
**Marital status**						
Married		1.00	1.00		1.00	1.00
Not married		1.13 (0.96–1.34)	1.05 (0.89–1.25)		1.07 (0.85–1.35)	0.99 (0.78–1.24)
Widowed		1.12 (0.98–1.28)	1.05 (0.92–1.20)		1.06 (0.88–1.27)	0.96 (0.80–1.15)
**Race/ethnicity**						
White (non-Hispanic)		1.00	1.00		1.00	1.00
Black (non-Hispanic)		0.59 (0.50–0.70)	0.52 (0.44–0.62)		0.52 (0.41–0.66)	0.44 (0.34–0.56)
Hispanic/Latino		0.74 (0.58–0.95)	0.75 (0.58–0.95)		0.82 (0.59–1.14)	0.85 (0.61–1.19)
Other		0.69 (0.51–0.94)	0.72 (0.53–0.98)		0.71 (0.47–1.06)	0.73 (0.49–1.09)
**Health-related covariates**						
Co-occurring conditions			1.25 (1.20–1.31)			1.47 (1.39–1.56)
Physical activity level			0.94 (0.90–0.97)			0.92 (0.87–0.97)
**Dizziness**						
No			1.00			1.00
Yes			1.63 (1.39–1.91)			2.00 (1.62–2.46)
**ADL difficulty**						
No			1.00			1.00
Yes			1.56 (1.44–1.69)			1.79 (1.61–1.98)

Abbreviations: cOR = crude odds ratios; aOR = adjusted odds ratio; ADL = Activities of Daily Living. Notes: **^1^** Adjusted for sociodemographic characteristics; **^2^** Adjusted for sociodemographic characteristics and physical/functional health characteristics.

**Table 3:** Neighborhood physical environment and odds of falls using mixed-effect logistic regression models (robust variance estimation), *n* = 9259.

Characteristics	Any Fall			Multiple Falls		
	cOR	aOR ^1^	aOR ^2^	cOR	aOR ^1^	aOR ^2^
**Neighborhood physical environment**	0.95 (0.92–0.98)	0.94 (0.91–0.97)	0.96 (0.93–0.99)	0.93 (0.89–0.97)	0.92 (0.88–0.96)	0.95 (0.91–1.00)
**Time (HRS wave)**	1.07 (1.05–1.09)	1.08 (1.06–1.10)	1.10 (1.07–1.12)	1.13 (1.10–1.16)	1.14 (1.11–1.17)	1.16 (1.13–1.19)
**Demographic characteristics**						
Years of education		0.99 (0.97–1.00)	1.01 (0.99–1.03)		0.97 (0.95–0.99)	1.00 (0.98–1.03)
Wealth (log-transformed)		0.65 (0.49–0.86)	0.90 (0.69–1.16)		0.62 (0.40–0.96)	1.06 (0.71–1.58)
**Age group**						
65–69		1.00	1.00		1.00	1.00
70–74		1.20 (1.05–1.37)	1.14 (1.00–1.30)		1.29 (1.07–1.55)	1.22 (1.01–1.46)
75–79		1.65 (1.42–1.91)	1.45 (1.25–1.67)		1.78 (1.46–2.19)	1.46 (1.19–1.78)
80–84		1.77 (1.49–2.11)	1.51 (1.27–1.80)		1.83 (1.44–2.33)	1.43 (1.12–1.81)
85 and older		2.40 (1.95–2.95)	1.82 (1.47–2.25)		2.85 (2.14–3.78)	1.95 (1.46–2.62)
**Sex**						
Male		1.00	1.00		1.00	1.00
Female		1.21 (1.08–1.35)	1.20 (1.08–1.34)		1.01 (0.87–1.17)	1.00 (0.86–1.16)
**Marital status**						
Married		1.00	1.00		1.00	1.00
Not married		1.13 (0.95–1.34)	1.05 (0.89–1.24)		1.08 (0.86–1.37)	0.99 (0.78–1.25)
Widowed		1.12 (0.98–1.28)	1.05 (0.92–1.20)		1.05 (0.88–1.27)	0.96 (0.80–1.15)
**Race/ethnicity**						
White (non-Hispanic)		1.00	1.00		1.00	1.00
Black (non-Hispanic)		0.59 (0.50–0.70)	0.52 (0.44–0.62)		0.52 (0.41–0.66)	0.44 (0.34–0.57)
Hispanic/Latino		0.74 (0.58–0.95)	0.74 (0.58–0.95)		0.83 (0.60–1.16)	0.86 (0.62–1.20)
Other		0.68 (0.50–0.93)	0.71 (0.52–0.97)		0.67 (0.44–1.01)	0.70 (0.47–1.04)
**Health-related covariates**						
Co-occurring conditions			1.26 (1.20–1.31)			1.48 (1.39–1.57)
Physical activity level			0.94 (0.90–0.97)			0.91 (0.87–0.96)
**Dizziness**						
No			1.00			1.00
Yes			1.63 (1.39–1.90)			2.00 (1.62–2.47)
**ADL difficulty**						
No			1.00			1.00
Yes			1.56 (1.44–1.70)			1.80 (1.62–2.00)

Abbreviations: cOR = crude odds ratio; aOR = adjusted odds ratio; ADL = Activities of Daily Living. Notes: **^1^** Adjusted for sociodemographic characteristics; **^2^** Adjusted for sociodemographic characteristics and physical/functional health characteristics.

**Table 4:** Neighborhood characteristics and adjusted odds of falls using mixed-effect logistic regression models (robust variance estimation), *n* = 9259.

Characteristics	Any Fall	Multiple Falls
	aOR	aOR
**Neighborhood social cohesion**		
I feel part of this area	0.95 (0.93–0.98)	0.95 (0.92–0.99)
I trust people in this area	0.98 (0.95–1.01)	0.96 (0.92–0.99)
People in this area are friendly	0.98 (0.95–1.01)	0.96 (0.93–1.00)
People in this area will help you	0.97 (0.94–1.00)	0.96 (0.93–1.00)
**Neighborhood physical environment**		
Absence of vandalism/graffiti	0.98 (0.96–1.01)	0.99 (0.95–1.02)
Absence of rubbish	0.97 (0.94–1.00)	0.95 (0.91–0.99)
Absence of vacant/deserted houses	0.98 (0.96–1.01)	0.98 (0.95–1.01)
Safe walking alone at night	0.97 (0.95–1.00)	0.97 (0.94–1.00)

Abbreviations: aOR = odds ratio, adjusted for demographic characteristics (education, wealth, age, sex, marital status, race/ethnicity) and physical/functional health-related covariates (co-occurring chronic health conditions, physical activity level, self-reported dizziness, and activities of daily living difficulty).
